# Setbacks in the road to self-injection: a descriptive study of provider and mystery client reports on the DMPA-SC care-seeking experience in Nigeria

**DOI:** 10.3389/fgwh.2025.1552379

**Published:** 2025-08-13

**Authors:** Madeline Griffith, Sneha Challa, Ayobambo Jegede, Ivan Idiodi, Chioma Okoli, Aminat Tijani, Shakede Dimowo, Awawu Grace Nmadu, Elizabeth Omoluabi, Jenny Liu

**Affiliations:** ^1^ Institute for Health & Aging, School of Nursing, University of California, San Francisco, CA, United States; ^2^ AkenaPlus Health, Abuja, Nigeria

**Keywords:** self-injection, self-care, DMPA-SC, implementation research, mixed-methods, refills, method continuation

## Abstract

This mixed-methods study describes perspectives from health providers and simulated clients on initiation and continuation of DMPA-SC for self-injection in Nigeria. Through mystery (simulated) client interactions, we found that providers were similarly willing to dispense units of DMPA-SC for self-injection to different client profiles, which varied in age, marital status, and parity. However, in-depth interviews with providers revealed nuance in their approaches to assessing clients’ eligibility for unsupervised self-injection of DMPA-SC. Factors including client age, marital status, parity, and education influenced who they deemed able to self-inject, which may limit access to DMPA-SC for clients who wish to self-inject the method. Quantitative and qualitative data also indicated that clients faced setbacks in continuing unsupervised self-injection when seeking refills from an unfamiliar provider. Stockouts of DMPA-SC further complicated access; some providers resorted to purchasing DMPA-SC privately and passing the cost onto clients. These findings highlight the need for clearer refill protocols and more consistent supply to ensure equitable access to DMPA-SC for self-injection in Nigeria.

## Introduction

The contraceptive method subcutaneous depot medroxyprogesterone acetate (DMPA-SC) offers users the option to self-inject DMPA-SC from a pre-filled syringe with a small needle. This self-care method gives individuals the ability to promote and maintain their own health with or without support from a health worker (1). Contraceptive self-care has the potential to increase choice and autonomy among individuals while decreasing the burden on health care providers (1, 2). Self-injectable contraception has also been touted as a promising method to improve contraceptive access, empowerment, and continuation rates^1^ among a broad range of clients, including young women who have been shown to experience provider bias and barriers to contraceptive services (3–6). This descriptive study relies on data from mystery (simulated) client visits and in-depth interviews with family planning providers to illustrate both provider and simulated client perspectives on initiating and continuing self-injectable DMPA-SC. Our aim was to track the continued scale-up of self-injectable DMPA-SC in Nigeria.

Self-injectable DMPA-SC was first implemented in select states and service delivery points (SDPs) in Nigeria in 2015, and scale-up to additional states and healthcare provider cadres has been underway since 2019. Our focus is on this “scale-up” phase—to understand how implementation can be optimized to meet client and provider needs as the method becomes more available. DMPA-SC was first introduced in Nigeria through private SDPs in ten South-Western states (7). As part of a total market approach to scale-up DMPA-SC for self-injection, distribution of the method in public SDPs began in 2016 (8). The Nigerian government has worked with implementing partner programs to train family planning providers, in both private and public SDPs, on the provision of DMPA-SC for self-injection. In this paper, private SDPs include pharmacies, patent and proprietary medicine vendors (PPMVs), and private hospitals/clinics. Private SDPs operate on a fee-for-service basis, where clients pay for both the contraceptive product and the provider's time spent on counseling or administering an injection. Public SDPs include government hospitals and clinics, which receive contraceptive products from the Nigerian government and provide them to clients at no cost. However, some facilities may charge a nominal fee for additional materials, such as gloves, alcohol wipes, and pregnancy tests.

Research has tracked the implementation and scale-up of DMPA-SC since the introduction of the method in Nigeria in 2015. Sieverding et al. first described the method initiation experience with a mystery client (MC) study in 2016 in South West Nigeria, which found provider bias in contraceptive provision to adolescents and young women (9). Despite the potential benefits of self-injection of DMPA-SC to empower self-care, persistent barriers hinder women's ability to initiate and continue using DMPA-SC, including the ability to reliably find and obtain refills. Structural barriers, including method availability, cost, and the multi-step dispensing guidelines, complicate method initiation and continuation for both providers and clients, as noted in Nigeria's National Guidelines for the Introduction and Scale-up of DMPA-SC Self-injection (10). Behavioral barriers, such as providers' judgments on method suitability for their clients and varied assessments of client eligibility for unsupervised self-injection, further restrict access for clients. For example, a qualitative analysis of interviews with healthcare providers in Uganda revealed their reservations about certain clients (e.g., younger, unmarried, and nulliparous clients) self-injecting DMPA-SC and indicate that they restricted access accordingly (11). In a mixed-methods study in South Africa and Zambia, some providers did not support or educate clients on self-care methods, including self-injection of DMPA-SC, sometimes out of concern that clients would not be able to correctly self-administer the injection (12). More broadly, providers have also been found to impose restrictions on giving hormonal contraception, including injectables (9, 10) to certain clients based on their own judgment rather than objective clinical assessment. Research has shown that pronatalist social norms, perceptions about the appropriateness of adolescent sexual activity, and concerns about the impact of hormonal contraceptives on fertility all contribute to biases against young and unmarried clients using contraception, which may impact this population's access to and continued use of contraception (13–15).

While the option for unsupervised self-injection of DMPA-SC offers users some flexibility and autonomy to administer the contraceptive themselves, users must still interact with the formal healthcare system for training and refills, presenting opportunities for access barriers to arise each time. For instance, the Nigerian Ministry of Health (MOH) outlined a multi-step process for providers to train clients on self-injection of DMPA-SC in the 2019 National Guidelines for the Introduction and Scale-up of DMPA-SC Self-injection (10). A provider should first train an interested client by demonstrating self-injection using visual aids or a model. The provider may then supervise the client's first self-injection of DMPA-SC. If the provider deemed the client competent with self-injection, “assessed based on correct performance of the four critical steps to self-injection”^2^, the client could have a follow-up visit with a provider for another supervised self-injection of DMPA-SC (10). After the second self-injected dose was performed under direct observation of a provider, the provider could dispense two doses of DMPA-SC for unsupervised self-injection. While the client must return to a provider to obtain more doses, there were no specific criteria for objectively assessing a client's competency to self-inject DMPA-SC, leaving the decision to the provider's discretion.

The potential benefits of self-injectable DMPA-SC to promote contraceptive self-care include increasing women's control in family planning and decreasing the time and cost of frequent clinic visits. So, implementation and scale-up of DMPA-SC for self-injection in Nigeria is being studied extensively to understand how well provision of the method has met the needs of clients and providers (10). To date, this work has often focused on the initiation of the method, and the likelihood and predictors of method continuation (5, 7, 16). However, there is little in-depth study of the experience of seeking (from the client perspective) and distributing (from the provider perspective) refills of DMPA-SC for self-injection. This analysis aims to address this gap by examining the DMPA-SC refill-seeking and dispensing experience in Lagos, Enugu, and Plateau states of Nigeria between November 2020 to May 2023, to identify facilitators and challenges to method continuation.

The research team adapted the methodology followed by Sieverding et al. in Lagos in 2020, which used two client profiles that differed in age, marital status, and parity, and added that the MC was interested in trying self-injectable DMPA-SC to the interaction script (17). The aim of this data collection activity was, in part, to assess whether simulated clients were being subjected to the same provider biases against young, unmarried, nulliparous clients seeking contraceptive services in Nigeria that have been previously documented in the literature (3, 9, 18). Consistent with Sieverding et al.'s findings, an analysis of this data led by Challa et al. found that younger, unmarried MC actors faced more scrutiny from providers when seeking DMPA-SC for self-injection, though actual dispensation rates of the method did not differ based on the actors' portrayed age (17). This paper expands the scope of Challa et al.'s analysis of the DMPA-SC initiation experience by incorporating MC data from two additional study sites in Nigeria, and provides new data on the refill-seeking experience for continued self-injection of DMPA-SC from these sites.

To ensure representation across socioeconomic status, religion, education, and other factors that influence contraceptive choice and use, the study sites were purposively selected from various regions of Nigeria: Lagos (South West), Plateau (North Central), and Enugu (South East) (19). We sampled from public and private SDPs in Lagos and Plateau, and only public SDPs in Enugu. At the time of data collection, the progress of implementation via the total market approach varied state by state; thus, the sampling of SDPs varied accordingly in our study. We described the experiences of mystery client (MC) actors who posed as clients interested in initiating or continuing DMPA-SC for self-injection, to better understand the care-seeking experience of those who use or would like to use this method for self-injection in three states of Nigeria. We further contextualized the process of initiating and continuing self-injection of DMPA-SC using interview data from providers in public and private SDPs who train clients on self-injection of DMPA-SC.

## Methods

We used MC actors to describe simulated client interactions with providers in public and private SDPs in three states of Nigeria. MC methodology allows client-provider interactions to be reported from the perspective of the simulated client, and has been used in many sexual and reproductive health studies (20). This method offers the advantage of controlling for client characteristics, presentation, and inquiries to the providers across many interactions.

We built on Sieverding et al.'s data collection methods from 2016, of MCs seeking contraception from providers in South West Nigeria, with a new sample and specification in the interaction script that the MC was seeking to start or continue self-injection of DMPA-SC, given the evolution of the national program. MC actors portrayed clients in two different care-seeking scenarios: (1) New user: actors posed as clients who want to start self-injecting DMPA-SC; (2) Continuing user: actors posed as DMPA-SC users seeking refills for unsupervised self-injection from SDPs they had not previously visited.

We supplemented these quantitative data from MC interactions with qualitative data from healthcare providers recruited from the same SDPs that MC actors visited. With quantitative data from the MC interactions, we described the availability of DMPA-SC in our sample of SDPs and directly compared the experience of accessing DMPA-SC for self-injection between the two simulated client profiles. Qualitative data from providers gave us the opportunity to describe nuance in how providers counseled on and distributed DMPA-SC for self-injection, evaluated clients' eligibility for unsupervised self-injection, and navigated challenges in training clients and dispensing this method. By describing the findings from both data collection activities together we aimed to give a more comprehensive picture of the implementation of DMPA-SC for self-injection than we would have provided with either dataset alone. This allowed us to describe both the measurable aspects of accessing the method and the real-life nuances that shaped client and provider experiences.

### Quantitative sample and data collection: mystery client interactions

Per methods described elsewhere (17), we worked with family planning implementing partner programs to identify three local government areas (LGAs) in Lagos state, and two LGAs each in Enugu and Plateau states, where providers had been trained on providing DMPA-SC for self-injection. At the time of data collection, implementation of DMPA-SC for self-injection was at different stages across the country (Table 1). Lagos was among the first states where DMPA-SC for self-injection was implemented among private retailers, and later included public sector provision as well. In Enugu, we sampled from public SDPs for new user MC visits and corresponding provider IDIs, and from public and private SDPs for continuing user MC visits and corresponding provider IDIs. Implementation of DMPA-SC for self-injection among private retailers in Enugu was not fully underway at the start of data collection (2022), so they were incorporated for continuing user MC visits approximately one year later (2023). Finally, implementing partner programs had only trained public sector providers in Plateau at the time of data collection, thus only public SDPs were sampled.

**Table 1:** Data collection timeline and stages of DMPA-SC^a^ for self-injection implementation.

Data collection activity	Dates of data collection (mystery client interactions; provider IDIs)	Sampling and status of DMPA-SC for self-injection implementation at time of data collection, by sector
“New user” mystery client interactions; provider IDIs	Lagos: November-December 2020; February 2021	Public and private SDPs^b^ sampled; DMPA-SC for self-injection implemented in both sectors.
	Enugu: July 2022; August 2022	Public SDPs sampled; private SDPs not yet trained on provision of DMPA-SC for self-injection.
	Plateau: July 2022; August 2022	Public SDPs sampled; private SDPs not yet trained on provision of DMPA-SC for self-injection.
“Continuing user” mystery client interactions; provider IDIs	Lagos: February-March 2022; June 2022	Public and private SDPs sampled; DMPA-SC for self-injection implemented in both sectors.
	Enugu: May 2023; November 2023	Public and private SDPs sampled; DMPA-SC for self-injection implemented in both sectors.
	Plateau: May 2023; November 2023	Public SDPs sampled; private SDPs not yet trained on provision of DMPA-SC for self-injection.

^a^ Subcutaneous depot medroxyprogesterone acetate.

^b^ Service delivery point.

We stratified the sampling frame of SDPs within each care-seeking scenario (new users v. continuing users) and state by LGA and sector. For new user interactions, we randomly selected 60 SDPs in Lagos (30 public, 30 private), 42 in Enugu (all public), and 67 in Plateau (55 public, 12 private). For continuing user interactions, we again randomly selected 60 SDPs in Lagos (30 public, 30 private), 42 in Enugu (all public), and 67 in Plateau.

**Figure 1:**
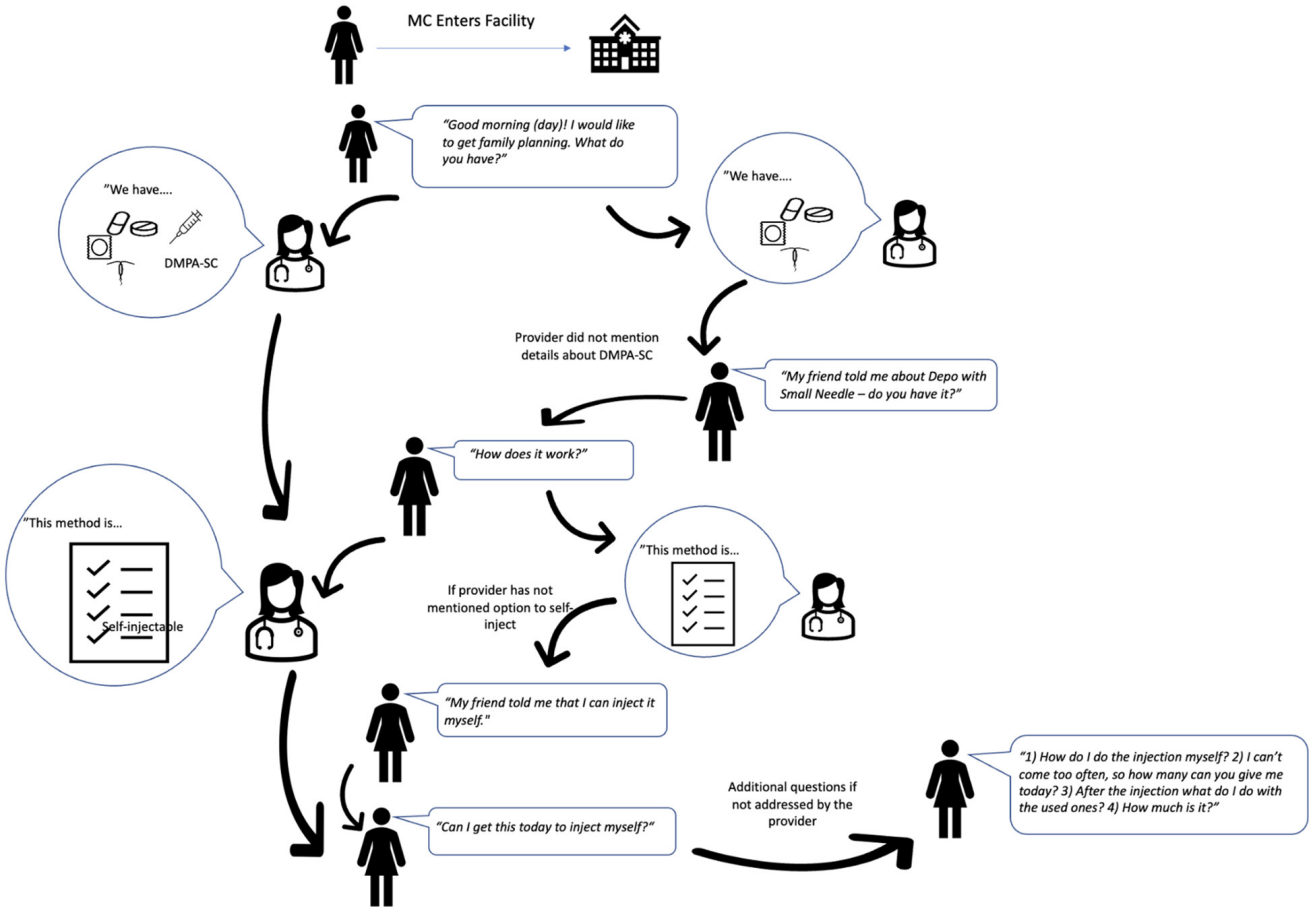
Mystery client flow diagram: new users of DMPA-SC for self-injection (17).

**Figure 2:**
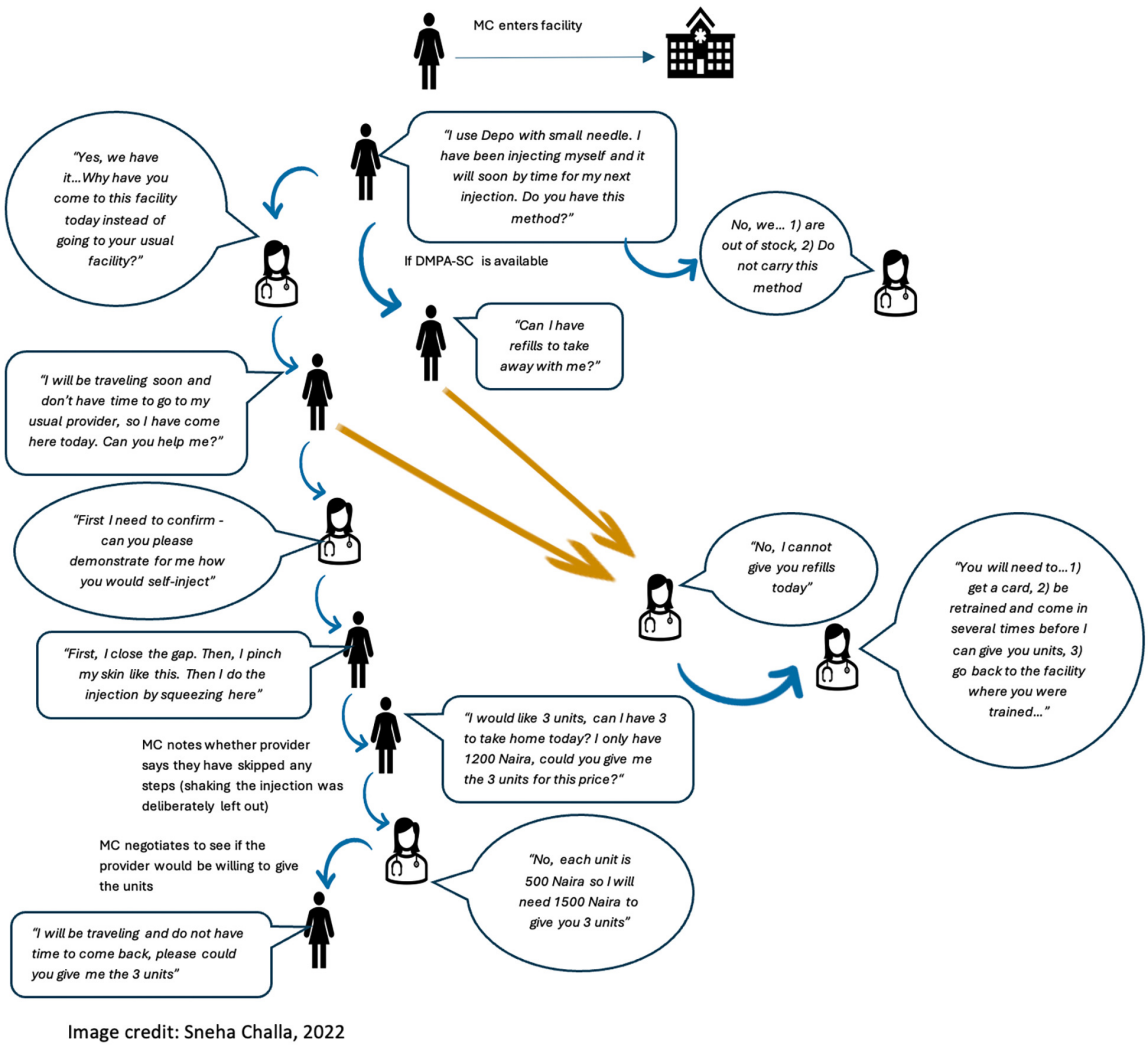
Mystery client flow diagram: continuing users of DMPA-SC for self-injection.

Twenty-four actors (8 in each state) were trained over five days to follow a script to seek counseling on self-injection of DMPA-SC, and units of DMPA-SC for unsupervised self-injection (interaction flow chart in Figures 1, 2). MC actors were selected from the research team's pool of interviewers based on their area of residence, ability to speak the local language fluently, and prior experience with MC interactions and data collection in general. Actors were trained to standardize their behavior across interactions, to dress age-appropriate for the client they were portraying, to allow conversation with the provider to progress naturally, and to not share personal details about their client profile unless specifically asked by the provider (e.g., their age, marital status, education, etc.). The training was followed by a day of pilot testing, where actors visited nearby SDPs and tested the script with real providers. Twenty-three of the same actors participated in new and continuing user MC visits, one actor who did new user visits in Lagos was replaced because she was unavailable for continuing user visits. In each scenario, actors portrayed one of two client profiles: (a) an 18–19-year-old unmarried woman with no children, or (b) a 30–31-year-old married woman with three children. Each profile visited each selected SDP once; visits were randomized by time of day (morning or afternoon) and by which profile visited first, with a minimum of two days between visits to the same SDP. New user actors asked family planning providers about which contraceptive options they had, and later stated that a friend told them about self-injectable DMPA-SC and that they wanted to try it themselves. Actors were not instructed to speak to a specific provider or type of provider within the SDP, but rather to allow the staff to direct them to an appropriate provider. New user visits were conducted over approximately two weeks in each study site: Lagos (November to December 2020), Enugu (July 2022), and Plateau (July 2022). Continuing user actors told providers they had been self-injecting DMPA-SC for 1.5 years and wanted to obtain refills of the method to continue self-injecting. Continuing user visits were conducted in Lagos (February-March 2022), Enugu (May 2023), and Plateau (May 2023).

After each visit, actors completed a debrief survey, which included questions on counseling for DMPA-SC, contraceptive method recommendations from the provider, and whether they obtained DMPA-SC for unsupervised self-injection. Actors also recorded a brief audio narrative noting their impression of the SDP, their perceptions of comfort and privacy, and reasons the provider would or would not train the actor on self-injection and/or give them units of DMPA-SC to take home for self-injection.

### Qualitative sample and data collection: in-depth interviews with family planning providers

**Figure 3:**
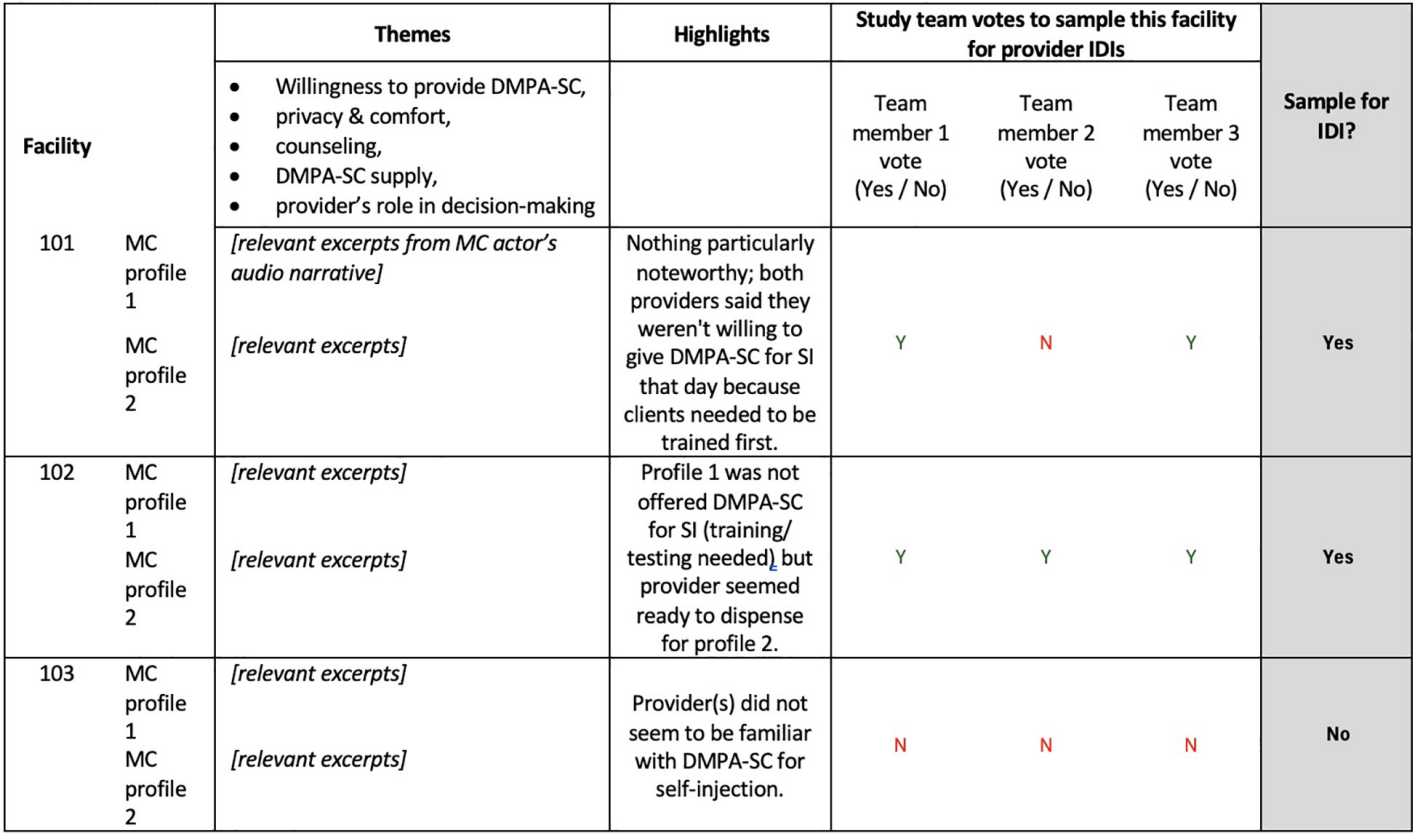
Template example for IDI sampling from MC audio narratives. Details of the MC interaction were extracted from transcribed audio narratives in the “Themes” column. Using the extracted excerpts, study team members summarized findings in the “Highlights” column. Study team members then independently voted on whether to sample the facility for provider IDIs.

Following MC interactions, we reviewed actors' post-interaction audio recordings to identify SDPs from which to recruit providers for in-depth interviews (IDIs). We first extracted details of the interaction from transcribed audio narratives in a spreadsheet matrix organized by SDP and client profile, including anything the actor deemed noteworthy about the interaction (Figure 3). We then purposively selected a subset of SDPs from which to recruit providers for IDIs to represent different MC interaction experiences, such as differential reports of privacy/comfort from the two client profiles within an SDP, and some SDPs where actors were given units of DMPA-SC for self-injection and some where they were not.

We conducted 141 IDIs with selected providers trained in counseling and provision of DMPA-SC for self-injection (Lagos *n* = 51, Enugu *n* = 45, Plateau *n* = 45). Multilingual researchers with prior experience conducting IDIs were trained on the semi-structured interview guides before each round of interviews. We conducted a first round of interviews following the new user MC interactions, and a second round of interviews following the continuing user MC interactions. Each round was completed over approximately two weeks in Lagos (February 2021 and June 2022), Enugu (August 2022 and November 2023), and Plateau (August 2022 and November 2023).

Interviewers obtained informed consent from providers and asked about their role in contraceptive method counseling, how the introduction of DMPA-SC for self-injection has affected their work, and their process for providing DMPA-SC for self-injection including assessing a client's eligibility to self-inject. Questions about counseling and provision of DMPA-SC for self-injection were further specified through two “vignettes” of hypothetical client profiles that matched the MC profiles: (1) an approximately 30-year-old married woman with children and (2) an approximately 18-year-old unmarried woman with no children. We asked providers whether they believed the clients in the vignettes were well-suited to self-inject DMPA-SC, and if and how they would counsel them on self-injection of DMPA-SC. Each IDI lasted about 60 min and was audio recorded, transcribed and simultaneously translated to English if necessary. For transcription quality assurance, each interview audio recording was reviewed by a different team member than the original interviewer.

### Analysis

To describe the more structural components of initiating and continuing self-injection of DMPA-SC, we used quantitative and qualitative data on method availability and contraceptive counseling, and the hypothetical ability of clients to obtain units of DMPA-SC for self-injection in line with national guidelines.

To describe the more behavioral aspects of method initiation and continuation, we used quantitative data from MC interactions to identify potential differentials in how older and younger profile actors were treated. This included examining providers' recommendations for and against specific contraceptive methods for different client profiles, and how providers assessed the MCs’ eligibility to be allowed to do unsupervised self-injection of DMPA-SC. To help contextualize results from the MC interactions, we supplemented these data with qualitative data from provider IDIs. We examined provider perspectives on offering DMPA-SC for self-injection in general and to the vignetted client profiles, and their process for assessing client eligibility to initiate or continue self-injection of DMPA-SC. Details on the survey questions and variables used in this analysis are in Table 2.

**Table 2:** Summary of variables and measurement descriptions.

Measure	Survey question	Measurement
Provider familiar with DMPA-SC	Did your provider know what Depo with small needle^a^ was?	Binary
Provider had DMPA-SC in stock	Did the provider have Depo with small needle available?	Binary. Relevant if the actor indicated that the provider was familiar with DMPA-SC.
Provider had heard of self-injection before	Had the provider ever heard of self-injection before?	Binary. Relevant if the actor indicated that the provider was familiar with DMPA-SC.
Provider expressed moral or religious judgment about contraception	Express any moral or religious judgments about you wanting to use contraception?	Binary
Provider agreed to give DMPA-SC unit for self-injection today	Did the provider agree to give you Depo with small needle to inject yourself today?	Binary. Relevant if the actor indicated that the provider had heard of SI before.
Number of contraceptive methods discussed	Which other types of contraception (aside from Depo with small needle) did the provider mention?	Actors selected all methods discussed and the total number was summed.
Provider described side effects of DMPA-SC	Did s/he describe side effects of Depo with small needle?	Binary. Relevant if the actor indicated that the provider was familiar with DMPA-SC.
Side effect counseling for methods aside from DMPA-SC	Did s/he discuss possible side effects for any of these other methods?	Binary. Relevant if the actor indicated that the provider discussed any other methods aside from DMPA-SC.
Side effects to dissuade contraceptive use	Did s/he use side effects as a way of dissuading you from using any/all contraceptives?	Binary
Contraceptive method recommended	Were there particular methods that the provider recommended to you?	Binary for each contraceptive method listed.
Contraceptive method recommended against	Were there particular methods that the provider specifically said was not recommended for you?	Binary for each contraceptive method listed.

^a^ “Depo with small needle” was used to indicate DMPA-SC in the mystery client debrief survey as it is a commonly known/colloquial term for the method.

Quantitative MC data were analyzed using Stata 17.0. Qualitative data were summarized in a phased analysis process from pre- to post-data collection, using a content analysis technique with deductive coding (21). We pre-defined code titles for each parent question from the interview guides, by which we organized interview data following transcription. We noted key observations pertaining to each title and extracted accompanying quotes, which we organized into a summary spreadsheet matrix (question titles as rows, respondents as columns). With interview observations and quotes organized in matrices, one matrix per state and round of interviews, we focused on the subset of titles that pertained to how providers assessed client eligibility to self-inject DMPA-SC, challenges those providers faced in such assessments, and various requirements that SDPs had for dispensing units of DMPA-SC.

### Ethical approval

As non-disclosure of the research activity is critical to the MC design, we did not obtain informed consent from providers that the MC actors interacted with. However, we did inform the implementing partner programs, who provided the sample frame of supported facilities, about the nature of the study. To protect the protocol of the MC study, even they were not informed of the facilities that would be visited. This and all other study activities were approved by the study's institutional review boards [University of California San Francisco Institutional Review Board (Study #20-32949), National Health Research Ethics Committee in Nigeria (Approval #NHREC/01/01/2007-25/09/2020)] (22).

## Results

Actors who portrayed new users seeking to initiate DMPA-SC for self-injection completed 378 interactions in Enugu (*n* = 108), Plateau (*n* = 153), and Lagos (*n* = 117), across 191 SDPs (*n* = 151 public, *n* = 40 private). Actors who portrayed continuing users seeking refills of DMPA-SC for self-injection completed 360 interactions in Enugu (*n* = 106), Plateau (*n* = 134), and Lagos (*n* = 120), across 180 SDPs (*n* = 140 public, *n* = 40 private). Approximately 80% of the SDPs visited were public, most providers in the sample were female (88%), estimated by actors to be 40–49 years old (42%), and were identified as clinicians by actors (69%) (Table 3).

**Table 3:** Mystery client interaction details^a^.

Variables		New users		Continuing users		Overall	
		(*n* = 378)		(*n* = 360)		Total (*n* = 738)	
		*N*	%	*N*	%	*N*	%
Mystery client (MC) profile	Older, married profile	190	50.3%	180	50.0%	370	50.1%
	Younger, unmarried profile	188	49.7%	180	50.0%	368	49.9%
Service delivery point (SDP) sector	Public	298	78.8%	280	77.8%	584	79.1%
	Private	80	21.2%	80	22.2%	154	20.9%
Provider gender	Female	322	85.2%	328	91.1%	650	88.1%
	Male	56	14.8%	32	8.9%	88	11.9%
Estimated provider age	Under 30	27	7.1%	49	13.6%	76	10.3%
	30–39	86	22.8%	102	28.3%	188	25.5%
	40–49	164	43.4%	146	40.6%	310	42.0%
	Over 50	101	26.7%	63	17.5%	164	22.2%
Provider type	Doctor, nurse/midwife	272	72.0%	235	65.3%	507	68.7%
	Pharmacist, chemist, patent & proprietary medical vendor (PPMV)	40	10.6%	50	13.9%	90	12.2%
	Community health worker/community-based distributor (CHW/CBD)	46	12.2%	36	10.0%	82	11.1%
	Other/did not say	20	5.3%	39	10.8%	59	8.0%
State	Enugu	108	28.5%	106	29.4%	214	29.0%
	Plateau	153	40.5%	134	37.2%	287	38.9%
	Lagos	117	30.9%	120	33.3%	237	32.1%

^a^ Data in Table 3 were reported by mystery client actors in their post-interaction debrief survey.

A total of 141 in-depth interviews were conducted with providers trained to dispense DMPA-SC for self-injection. Most interviewed providers were female (84%), and a plurality were community health practitioners in public SDPs (34%) (Table 4).

**Table 4:** Provider participant characteristics^a^.

Provider Interviews (*N* = 141)			
Variables		*n*	%
State	Lagos	51	36.2
	Enugu	45	31.9
	Plateau	45	31.9
Gender	Female	118	83.7
	Male	23	16.3
Type of provider	Community Health Practitioner^b^ in a PHC	48	34.0
	Pharmacist or PPMV^c^	36	25.5
	Nurse or midwife in a public health clinic	33	23.4
	CHP in private clinic, pharmacy, or PPMV	10	7.1
	Nurse or midwife in private clinic, pharmacy, or PPMV	8	5.7
	Officer in Charge^d^	6	4.3

^a^ Data were reported by providers at the start of their interview.

^b^ Community Health Practitioners (CHPs) are trained and licensed healthcare workers who deliver primary health care particularly in rural areas (28, 29). Community-based distributors (CBDs), community health extension workers (CHEWs), and community outreach resource personnel (CORP) are all types of CHPs.

^c^ PPMVs sell pharmaceutical products for profit and are not formally trained in pharmaceutical practice.

^d^ An Officer in Charge (OIC) manages the operations of a primary health facility. They may be nurses, midwives, CHEWs, Community Health Officers, etc.

### Dispensation rates of DMPA-SC for self-injection were similar between mystery client profiles, but provider discretion and inconsistent method availability decreased accessibility

**Figure 4:**
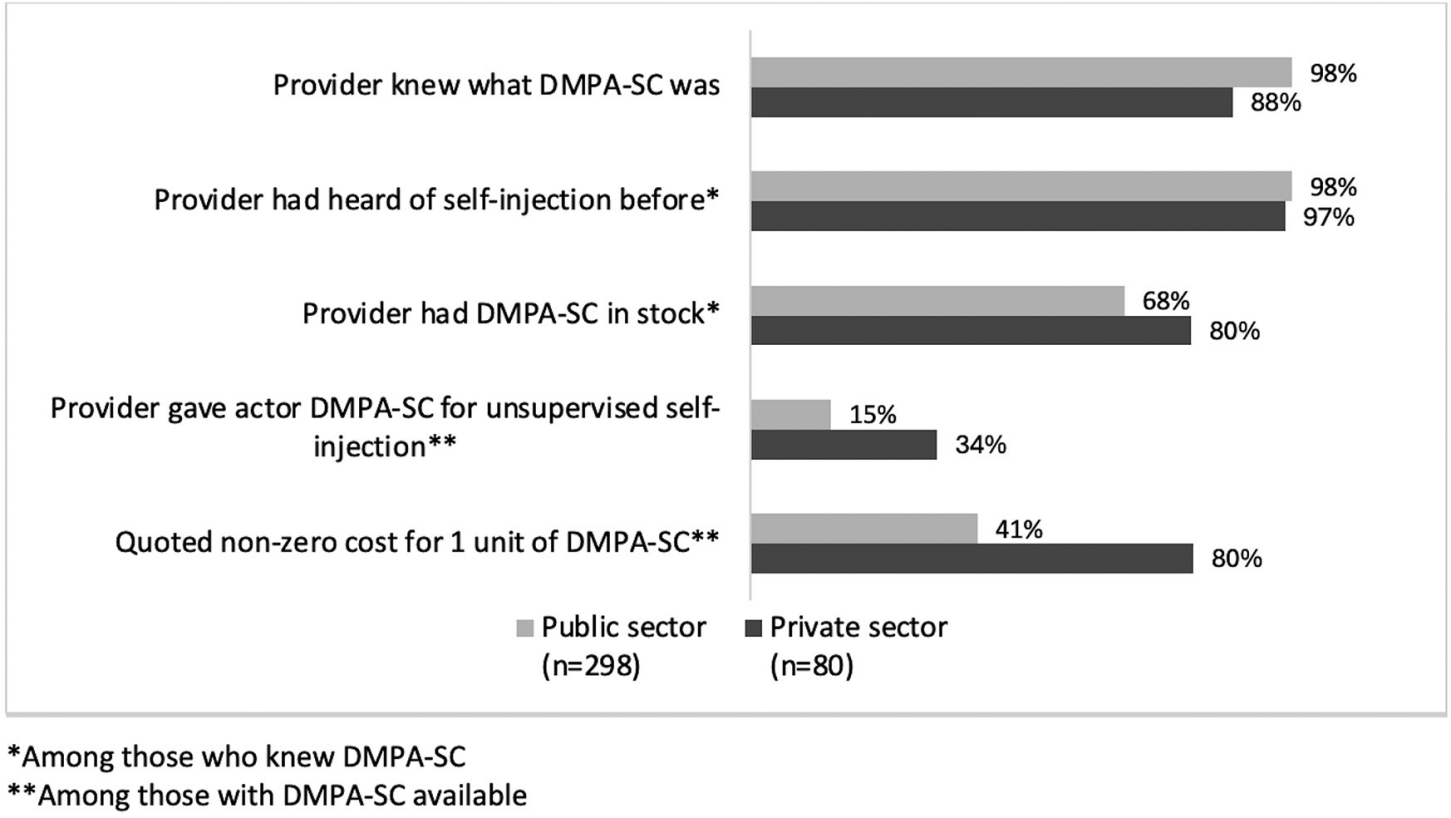
New user interactions: DMPA-SC availability and dispensation by SDP sector.

**Figure 5:**
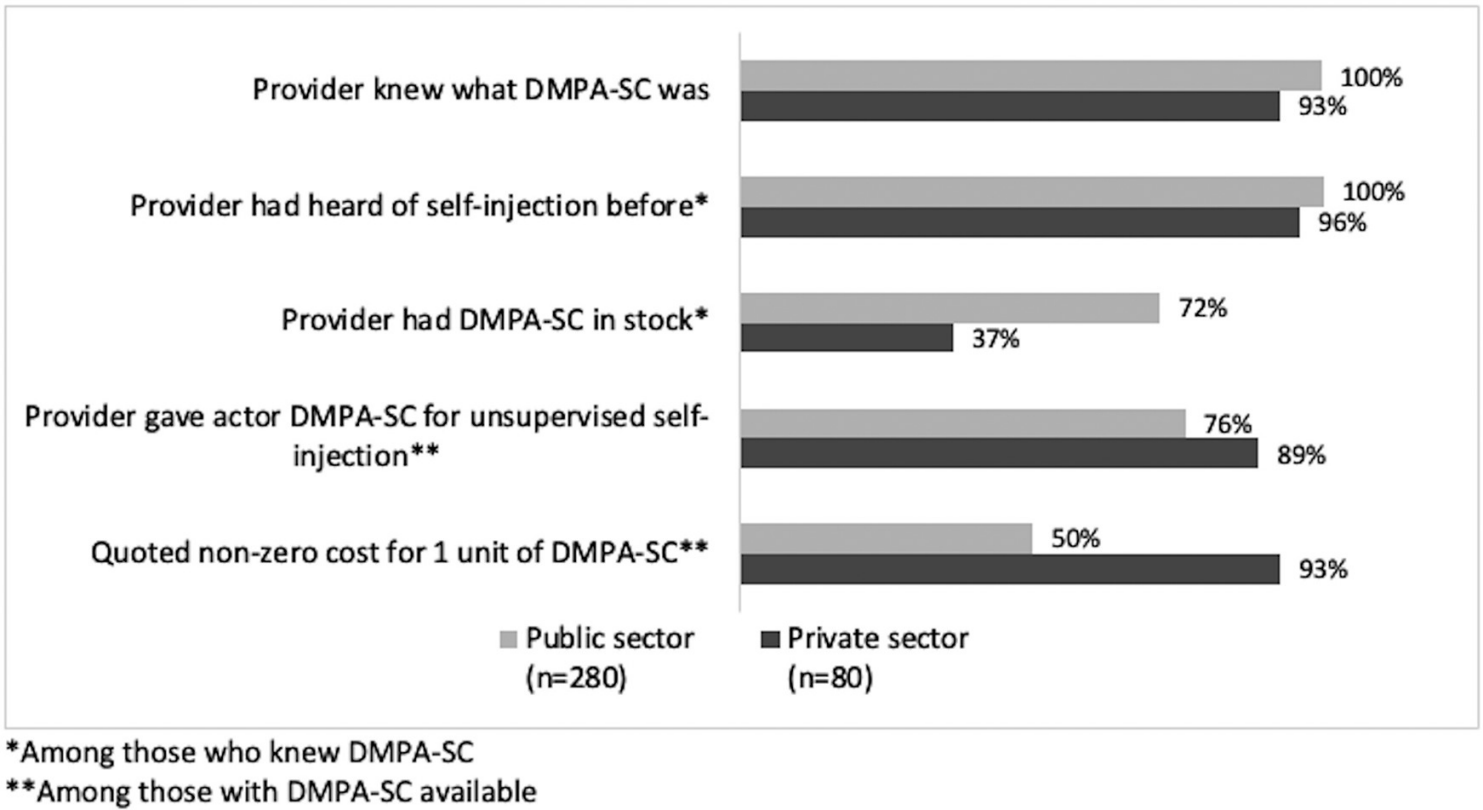
Continuing user interactions: DMPA-SC availability and dispensation by SDP sector.

Providers were familiar with DMPA-SC and the option for self-injection in nearly all MC interactions. However, method availability varied across states and SDP sectors. In new user interactions, DMPA-SC was in stock at only 67% of SDPs overall; a higher percentage of private SDPs had DMPA-SC in stock (80%) compared to public SDPs (68%) (Figure 4; Table 5). In continuing user MC visits, which occurred one to two years after new user MC visits, DMPA-SC availability was higher in public SDPs (72%) compared to private SDPs (37%) (Figure 5; Table 6).

**Table 5:** DMPA-SC dispensing details, by mystery client profile.

DMPA-SC dispensing details	New users (*N* = 378)				Continuing users (*N* = 360)				Overall (*N* = 738)					
	Younger, unmarried client profile (*n* = 188)		Older, married client profile (*n* = 190)		Younger, unmarried client profile (*n* = 180)		Older, married client profile (*n* = 180)		Younger, unmarried client profile (*n* = 368)		Older, married client profile (*n* = 370)		All interactions (*n* = 738)	
	*n*	%	*n*	%	*n*	%	*n*	%	*n*	%	*n*	%	*n*	%
Provider knew what DMPA-SC was	184	97.9%	179	94.2%	176	97.8%	177	98.3%	360	97.8%	356	96.2%	716	97.0%
Provider had heard of self-injection before (among those who knew DMPA-SC)	180	97.8%	176	98.3%	175	99.4%	175	98.9%	355	98.6%	351	98.6%	706	98.6%
Provider had DMPA-SC in stock (among those who knew DMPA-SC)	130	70.7%	125	69.8%	117	66.5%	111	62.7%	247	68.6%	236	66.3%	483	67.5%
Provider agreed to give DMPA-SC unit for unsupervised self-injection (among those with DMPA-SC available)	27	20.8%	21	16.8%	92	78.6%	85	76.6%	119	48.2%	106	44.9%	225	46.6%
Quoted non-zero cost for 1 unit of DMPA-SC (among those with DMPA-SC available)	55	42.3%	72	57.6%	70	59.8%	56	50.5%	125	50.6%	128	54.2%	253	52.4%
Median, mean cost per unit (among those quoted a non-zero cost)	500NGN^a^, 627NGN		500NGN, 506NGN		300NGN, 397NGN		400NGN, 396NGN		350NGN, 498NGN		400NGN, 458NGN		400NGN, 478NGN	
Number of DMPA-SC units provider agreed to dispense (among those who agreed to give DMPA-SC for unsupervised self-injection)														
1	9	33.3%	6	28.6%	29	31.5%	20	23.5%	38	31.9%	26	24.5%	64	28.4%
2	8	29.6%	2	9.5%	14	15.2%	20	23.5%	22	18.5%	22	20.8%	44	19.6%
>2	6	22.2%	8	38.1%	44	47.8%	42	49.4%	50	42.0%	50	47.2%	100	44.4%
Missing data	4	14.8%	5	23.8%	5	5.4%	3	3.5%	9	7.6%	8	7.5%	17	7.6%

^a^ NGN: Nigerian Naira, the currency of Nigeria. With average exchange rate in 2023, 500NGN was approximately $0.78 USD.

**Table 6:** DMPA-SC dispensing details, by service delivery point sector.

DMPA-SC dispensing details		New users (*N* = 378)				Continuing users (*N* = 360)				Overall (*N* = 738)					
		Public sector interactions (*n* = 298)		Private sector interactions (*n* = 80)		Public sector interactions (*n* = 280)		Private sector interactions (*n* = 80)		Public sector interactions (*n* = 578)		Private sector interactions (*n* = 160)		All interactions (*n* = 738)	
Provider knew what DMPA-SC was		293	98.3%	70	87.5%	279	99.6%	74	92.5%	572	99.0%	144	90.0%	716	97.0%
Provider had heard of self-injection before (among those who knew DMPA-SC)		288	98.3%	68	97.1%	279	100.0%	71	95.9%	567	99.1%	139	96.5%	706	98.6%
Provider had DMPA-SC in stock (among those who knew DMPA-SC)		199	67.9%	56	80.0%	201	72.0%	27	36.5%	400	69.9%	83	57.6%	483	67.5%
Provider agreed to give actor DMPA-SC unit for unsupervised self-injection (among those with DMPA-SC available)		29	14.6%	19	33.9%	153	76.2%	24	88.9%	182	45.5%	43	51.8%	225	46.6%
Quoted non-zero cost for 1 unit of DMPA-SC (among those with DMPA-SC available)		82	41.2%	45	80.4%	101	50.2%	25	92.6%	183	45.8%	70	84.3%	253	52.4%
Median, mean cost per unit (among those quoted a non-zero cost)		300NGN, 518NGN		500NGN, 633NGN		300NGN, 368NGN		512NGN, 494NGN		300NGN, 435NGN		500NGN, 590NGN		400NGN, 478NGN	
Number of DMPA-SC units provider agreed to dispense (among those who agreed to give DMPA-SC for unsupervised self-injection)															
1		10	34.5%	5	26.3%	40	26.1%	9	37.5%	50	27.5%	14	32.6%	64	28.4%
2		8	27.6%	2	10.5%	28	18.3%	6	25.0%	36	19.8%	8	18.6%	44	19.6%
>2		8	27.6%	6	31.6%	77	50.3%	9	37.5%	85	46.7%	15	34.9%	100	44.4%
Missing data		3	10.3%	6	31.6%	8	5.2%	0	0.0%	11	6.0%	6	14.0%	17	7.6%

In IDIs, respondents reflected on the challenges of unreliable supply of DMPA-SC, which included damaging the SDP's reputation as a reliable source of care, adding workload to the providers to source the product themselves, and negative effect on clients' continuity of care and affordability of the method:

“We are trained [to provide DMPA-SC for self-injection] but products are not available for us but are available in the open market.. I have spent my time and money and struggled to get [the] product in the open market and then they ask me to give them a report on what they could have provided? The supply chain of [DMPA-SC] has to be re-tailored in a way that practitioners will not have to depend on businessmen to get their supply.” (Male pharmacist in Lagos)

“They should make [DMPA-SC] readily available*.* […] when you introduce people to something and they begin to like it…if you withdraw it, they will start complaining a lot…they will say they are already used to DMPA-SC and don't want anything that will disorganize their body.” (Female nurse midwife in public clinic)

Actors were given at least one unit of DMPA-SC for unsupervised self-injection in 31% of new user visits, and 78% of continuing user visits, among visits where DMPA-SC was reportedly available. Across both new and continuing user visits, dispensation rates were higher among private SDPs compared to public, but were similar by client profile. Among the visits where continuing user actors reported that DMPA-SC was available, (*n* = 228), 55% were quoted some cost for the unit(s); the proportion of younger profile actors who were quoted a cost (60%) was higher than that of older profile actors (51%). When DMPA-SC was available and providers agreed to unsupervised self-injection, actors reported that providers would dispense between one to six units, and most providers agreed to dispense more than two units (Tables 4, 5).

**Figure 6:**
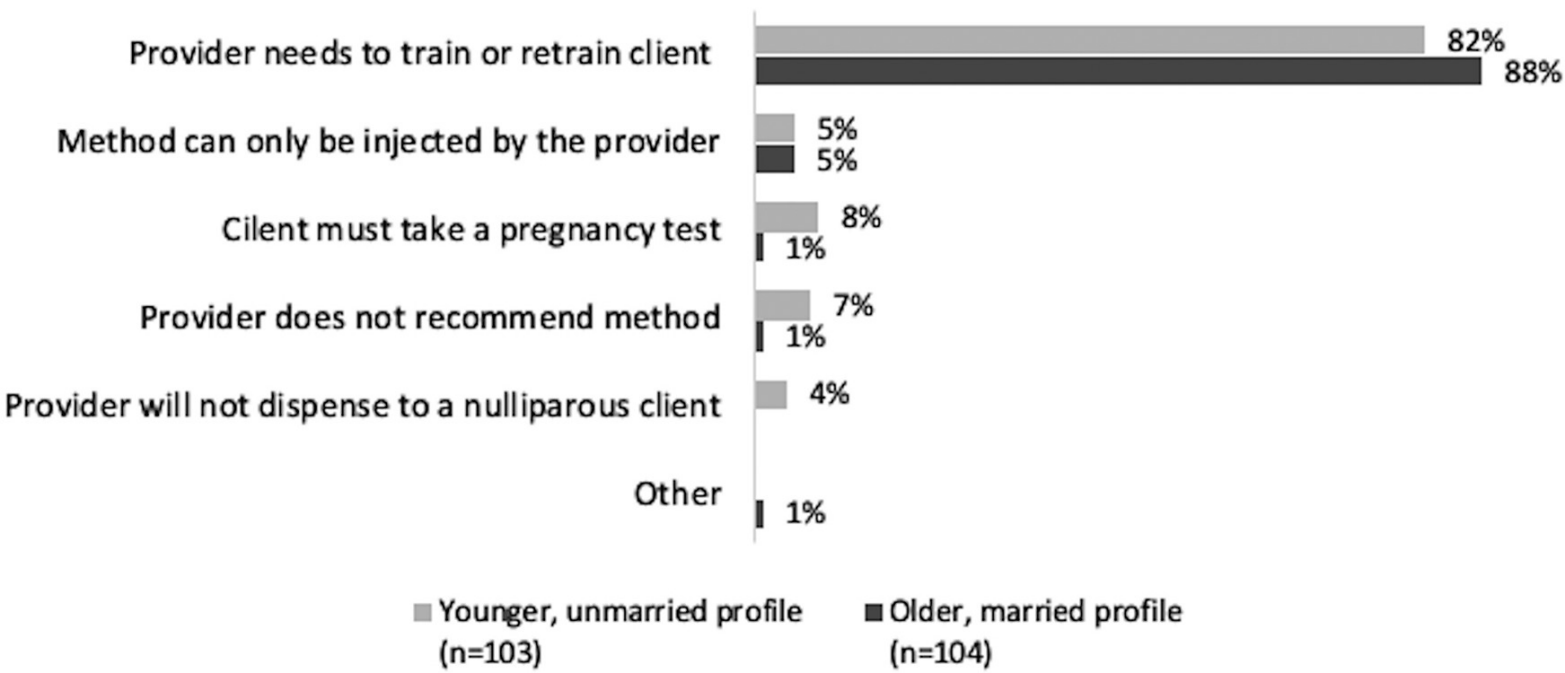
New user interactions: reasons provider did not agree to give actor a unit of DMPA-SC for unsupervised self-injection, by client profile.

In new user MC interactions where DMPA-SC was in stock and providers did not agree to dispense a unit to actors (*n* = 207), the most frequently cited reason was that the provider needed to train the MC on self-injection. A small number of actors reported other reasons they did not receive DMPA-SC for unsupervised self-injection; notably, in younger profile MC visits, actors reported they were told that they must take a pregnancy test first (8%), the provider did not recommend the method (7%), and the provider would not dispense DMPA-SC to a nulliparous client (4%). In contrast, older profile actors seldom reported these reasons (Figure 6).

In IDIs, respondents described how they have determined how many units of DMPA-SC to dispense to a client for unsupervised self-injection. Some respondents rationed the number of DMPA-SC units they dispensed to retain supply of the method for other clients. This female clinical health extension worker (CHEW/CHW) at a public facility in Enugu noted:

“When I consider the number remaining, I would give two [units of DMPA-SC for unsupervised self-injection]. It depends on the quantity available. If it is many, I may give three.”

Several IDI respondents have considered where clients live relative to the SDP, and dispensed more units of DMPA-SC for unsupervised self-injection to those who lived farther away, and fewer or none to those who lived closer:

“If their place is far, I give them 2 or 3 depending on the distance. If their places are close, I will give one so that I can monitor them.” (Female CHEW in a public clinic in Enugu)

“A client who is living close to me or close to the facility…there would be no need for self-injection. I would prefer to be administering [DMPA-SC] myself.” (Male CHEW in a public health clinic in Enugu)

Other respondents expressed concern that a client may give away a unit to someone else if they receive more than one unit of DMPA-SC at a time, as experienced by this female pharmacist in Enugu:

“Sometimes why I don't really sell [DMPA-SC] much to them is that I know of someone that I have given three [units]..But then she went and recommended it for a friend and gave her one when she doesn't know whether the person is actually fit to take it.”

Actors also reported on the cost of DMPA-SC units in interactions where the method was available. In new user interactions, most private SDPs (80%) charged for DMPA-SC, and 41% of public SDP interactions involved some cost. In continuing user interactions, most private SDPs (93%) quoted a cost for the method, and approximately half of public sector facilities (50%) did as well (Table 6). The median cost per unit by sector was approximately the same across new and continuing user interactions: 300 Nigerian naira (NGN) (approximately $0.47 USD in 2023) in public SDPs, and approximately 500NGN (approximately $0.78 USD in 2023) in private SDPs.

When asked about cost for contraceptives, most public sector respondents reported they are given free of charge. However, a few noted that stockouts of DMPA-SC that is typically provided free from the local government have led them to purchase the method themselves from another drug shop or market, and these costs of shoring up supply gaps were passed on to clients vis-à-vis a below-market rate fee:

“[Clients pay] 500 naira [for one unit of DMPA-SC] …because at times we go out of stock, we go to the pharmaceutical store to buy.” (Female CHEW at a private clinic in Plateau)

“There was a time that we ran short of the commodity and had to go outside to get and that made us charge our clients 200 naira for it.” (Female CHEW in a private clinic in Plateau)

Some participants from private SDPs reported a willingness to negotiate the price of DMPA-SC based on the client's ability to pay and their own preference to give the client something rather than nothing, such as this PPMV owner in Lagos:

“[Profit] matters but the positive effect on my community matters more to me because if they are not on family planning, they will visit quacks to do abortions. I decided to bring the cost down in my community so that women can have access to it..I do not want them to go away because of a huge cost.”

### Respondents' assessments of client eligibility to receive refills of DMPA-Sc for self-injection varied, sometimes setting clients back in the process to unsupervised self-injection, and often favoring educated and older individuals

Respondents described many ways of determining eligibility for the vignetted clients to continue with unsupervised self-injection of DMPA-SC. Most assessments fell into one of three categories: 1) verbal confirmation or demonstration of self-injection procedure; respondents stated they would ask the client to verbalize or demonstrate how she self-injects DMPA-SC without administering an active dose, such as this female CHEW in Enugu:

“I usually ask them to tell me how they used it. I will make her to show me how she does it at home. I will listen to be sure that she got the procedure.”

2) provider-supervised self-injection of DMPA-SC: many respondents said they have asked clients to self-inject DMPA-SC under their direct observation before dispensing units for the client to take home, meaning clients could seek refills only when they are due for a dose of DMPA-SC.

“They have to [self-inject] because if they cannot give themselves in my presence, definitely, they won't be able to give themselves at home.” (Female nurse at a public clinic in Lagos)

Or 3) provider-administered DMPA-SC; many respondents preferred to inject the client themselves, before the client could return for a future visit to continue in the stepwise self-injection initiation process.

“I would give her the injection by myself to refresh her memory.” (Male CHEW at a public clinic in Enugu)

Some respondents from private SDPs noted a higher profit potential from provider-administered DMPA-SC over dispensing DMPA-SC for client self-injection, as they charge an injection fee in addition to the cost of the method:

“We gain more by administering it because we sell [DMPA-SC], and we also charge for administering it.” (Female CHEW in Plateau)

**Figure 7:**
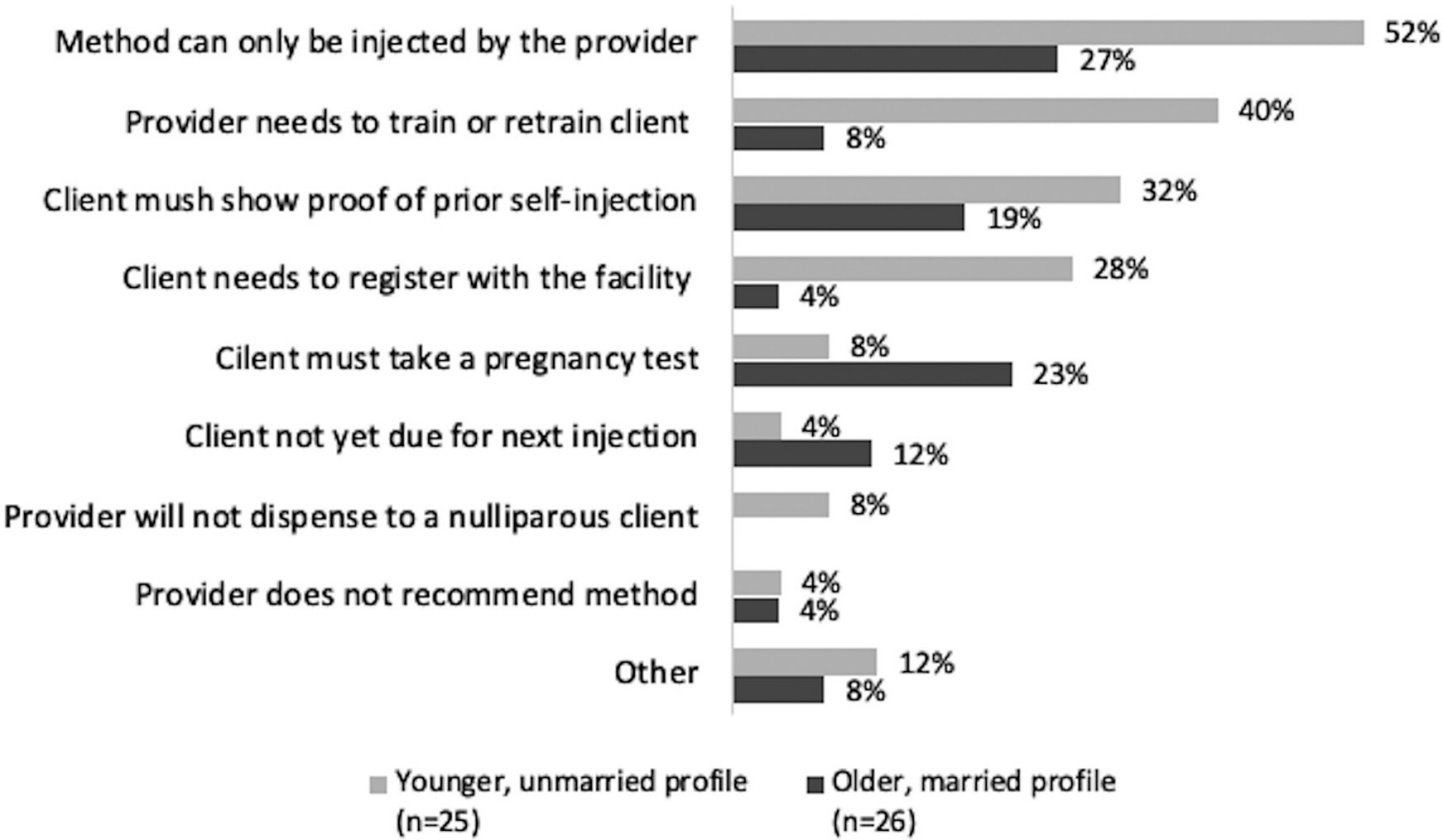
Continuing user interactions: reasons provider did not agree to give actor a unit of DMPA-SC for unsupervised self-injection, by client profile.

In continuing user MC interactions where DMPA-SC was in stock and providers did not agree to give the actor a refill unit (*n* = 51), the most common reasons providers cited were that at least the first injection at a given SDP had to be injected by the provider (39%), the provider required “proof” of prior self-injection (25%), and the provider needed to retrain the client on self-injection (24%). Younger profile actors were less frequently allowed to do unsupervised self-injection; 40% reported that the provider had to administer at least one, if not all, future doses to them. Figure 7 differentiates the reasons for not giving a refill unit by MC profile.

In IDIs, some respondents noted concerns about clients' ability to self-inject correctly and on time. In particular, several respondents felt that self-injection could only be offered to “educated” or “elite” clientele:

“[Offering self-injection] depends on their level of intelligence…In fact, there was one I gave to take home by the time she came back, she told me that, ‘mummy, I couldn't give it to myself’ that she wasted it. So, I started giving it to her by myself. Normally, I give one but there is this other woman…she is educated, she just buys and goes and gives to herself.” (PPMV in Lagos)

“Number one [who I recommend self-injection to] will be the elites in the society because they will understand better when they hear self-injection. What you will hear from the non-elites will be ‘no I don't like injection and you are talking about to inject myself’, but the former will like to know how it works and will want to access it.” (Male pharmacist in Enugu)

However, the appraisal of education and ability to self-inject was not a concern for all respondents interviewed, as this male CHEW in Plateau stated:

“[DMPA-SC] is very easy to administer, and every woman can learn it whether educated or not. They can do it because it is not difficult to administer.”

Other respondents preferred administering the injection themselves instead of clients self-injecting DMPA-SC, and typically cited a desire to maintain fidelity, and to protect client safety in the case of covert users:

“I will want them to come so I can administer it myself because I am not sure if they will administer it correctly.” (Female nurse in Plateau)

“We did not give her the self-injection to take home because maybe her husband will see it at home. […] When her date is due…she will just trek and come and meet us.” (Female CHEW in Plateau)

Despite these hesitations, many respondents noted the benefits that self-injection offers to both them and to their clients:

“[Self-injection of DMPA-SC] can benefit both staff and clients. For the staff it makes work easier and for the clients it gives her a sense of belonging that it is good for her, and she is part of it, so she can know how to use it for her family's good.” (Nurse in Plateau)

### Providers recommended injectables more to older profile actors, and episodic methods more to younger profile actors. Some cited contraceptive side effects to dissuade younger profile actors from using contraception

**Figure 8:**
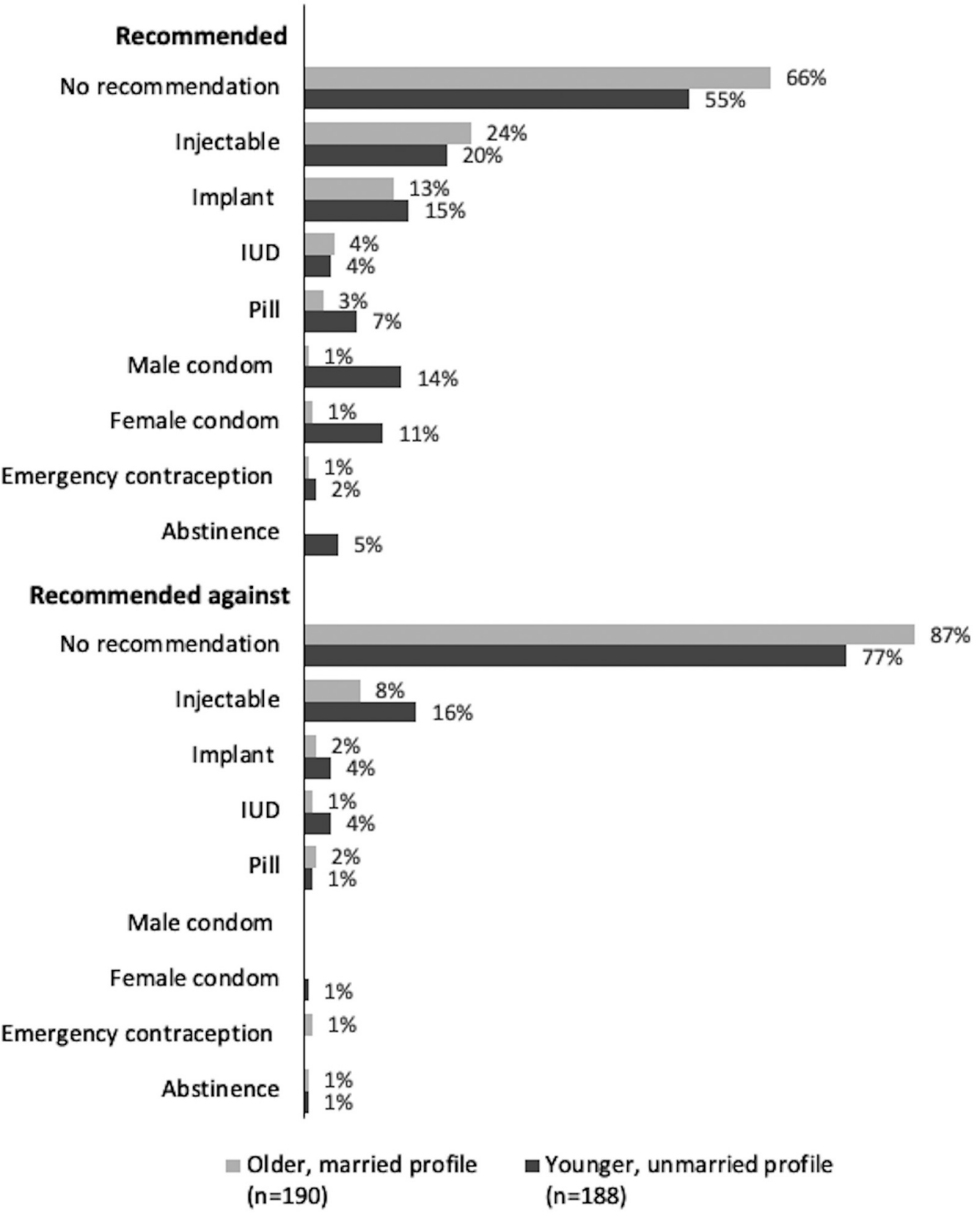
Providers’ recommendations for and against contraceptive methods in new user interactions, by mystery client profile.

In most new user MC interactions, providers discussed four or more contraceptive methods with the actor (64%, Table 7). Actors reported which contraceptive methods providers recommended and advised against, summarized by MC profile in Figure 8. Providers more frequently made a recommendation for a method to the younger, unmarried profile actors compared to older, married profile actors (45% and 34%, respectively). Similarly, 23% of younger profile actors were advised against at least one method, compared to 13% of older profile actors. Several methods were recommended to a similar proportion of both profiles, but the pill and male and female condoms were more frequently recommended to the younger profile, while injectables were more frequently recommended to the older profile. The largest difference in recommendations against a method between the two profiles was for injectables: 16% of younger profile actors and 8% of older profile actors were advised against using injectables.

**Table 7:** Contraceptive counseling experience, by mystery client profile.

Details of contraceptive counseling experience		New users (*N* = 378)				Continuing users (*N* = 360)				Overall (*N* = 738)					
		Younger, unmarried client profile (*n* = 188)		Older, married client profile (*n* = 190)		Younger, unmarried client profile (*n* = 180)		Older, married client profile (*n* = 180)		Younger, unmarried client profile (*n* = 368)		Older, married client profile (*n* = 370)		All interactions (*n* = 738)	
		*n*	%	*n*	%	*n*	%	*n*	%	*n*	%	*n*	%	*n*	%
Number of contraceptive methods discussed	0	8	4.3%	28	14.7%	118	65.6%	156	86.7%	126	34.2%	184	49.7%	310	42.0%
	1	6	3.2%	11	5.8%	28	15.6%	18	10.0%	34	9.2%	29	7.8%	63	8.5%
	2 to 3	37	19.7%	46	24.2%	24	13.3%	3	1.7%	61	16.6%	49	13.2%	110	14.9%
	4+	137	72.9%	105	55.3%	10	5.6%	3	1.7%	147	39.9%	108	29.2%	255	34.6%
Provider described side effects of DMPA-SC (among those who knew DMPA-SC)		85	46.2%	47	26.3%	*Not asked*		*Not asked*		N/A		N/A		N/A	
Provider counseled on side effects for methods aside from DMPA-SC (among those who discussed other methods)		71	29.4%	53	32.7%	11	17.2%	1	4.2%	82	33.9%	54	29.0%	136	31.8%
Provider cited side effects to dissuade contraceptive use		38	20.2%	22	11.6%	15	8.3%	0	0.0%	53	14.4%	22	5.9%	75	10.2%
Provider expressed judgment about client using contraception		29	15.4%	6	3.2%	18	10.0%	0	0.0%	47	12.8%	6	1.6%	53	7.2%

Across all interactions, MC actors reported that providers rarely cited negative side effects to dissuade contraceptive use (10%) or expressed judgment about them using contraception (7%). However, when such interactions occurred, these observations were more frequently reported by the younger, unmarried profile actors (14% and 13%, respectively), than by the older married profile actors (6% and 2%) (Table 7).

In IDIs, respondents shared their views on the appropriateness of various methods of contraception, including DMPA-SC, for their clientele. While many expressed that DMPA-SC is suitable for anyone who is interested, some voiced concerns about trusting the younger client vignette to manage self-injection, the risk of negative effect of hormonal contraception on fertility, and general hesitation to support contraceptive use for young, unmarried, and/or nulliparous clients.

“For self-injection, no. I don't trust [a 17-year-old girl], she can go and give [a DMPA-SC unit] to another person.” (Female nurse in public clinic, Lagos)

“This is someone that has not given birth before and this injection has to do with hormones, so I will not want something that will alter her from giving birth when she wants… So, what I will do is encourage her and educate her on the dangers it will cause her, and if she insists that she wants to take the injection, I will give it to her.” (Female CHEW in Plateau)

## Discussion

This study aimed to describe the experiences of initiating and continuing self-injection of DMPA-SC in Nigeria, provide insights into the status of implementation thus far, and highlight areas where implementation was not meeting the needs of clients or providers. We used quantitative data from MC interactions and qualitative interview data from family planning providers, collected at several timepoints during implementation of DMPA-SC for self-injection and in varied geographic regions of the country. This approach provided a range of perspectives on experiences accessing and providing DMPA-SC for self-injection in Nigeria.

The family planning providers we interviewed were recruited from the pool of SDPs that MC actors visited, which allowed for a more nuanced examination of care-seeking and provision of DMPA-SC for unsupervised self-injection than either data source could provide alone. The MC design allowed for a client-centered evaluation of interactions between providers and simulated clients, minimizing social desirability and response biases that can occur with self-reports from providers (18, 23). Further, though MC methodology does not eliminate reporting bias from the actors, it was likely reduced by having a small number of trained actors complete and report on many interactions. These observations offer a novel contribution to the literature on the implementation and scale-up of DMPA-SC for self-injection by including perspectives from both providers and simulated clients on method continuation and the refill-seeking experience, a shortcoming noted in other research (24).

We found mixed perceptions on which clients were well-suited to try unsupervised self-injection of DMPA-SC, and varied means of assessing client eligibility to continue unsupervised self-injection of the method. For example, some providers expressed concern that clients with low levels of formal education would not be able to self-inject the method. Other client demographics, including age, marital status, and parity, were not evaluated unidirectionally by respondents; some noted that unsupervised self-injection was better suited to “older” clients, while a couple of respondents thought that unsupervised self-injection was better for “younger” clients. Some respondents felt strongly that clients who were married and had given birth were more fit to try DMPA-SC for self-injection, though it was not always clear whether that perception was in reference to the hormonal method (DMPA-SC), or to the mode of administration (unsupervised self-injection). These findings are reminiscent of prior research on younger and unmarried clients facing provider-imposed restrictions to accessing self-injectable contraception out of concern for their ability to safely self-inject the method (11). Also, generally consistent with prior similar research on contraceptive method recommendations, the two MC profiles reported differing contraceptive method recommendations from providers (9). Injectables were more frequently recommended to older, married profile actors, and episodic methods were more frequently recommended to younger, unmarried profile actors. While the effect of provider influence on contraceptive access and decision-making has been documented for contraceptives in general, it is important to consider such influence in the context of self-injectable contraception (25, 26). Although self-care methods have the potential to increase client autonomy, clients must still interact with and rely on healthcare providers to access these methods, and provider biases and influence can compromise the potential for increased agency among users of these methods (1).

Most MC actors portraying continuing users were given or sold at least one refill unit of DMPA-SC, which suggests that many clients who choose to self-inject can continue the method without repeating steps in the self-injection initiation process. However, providers' self-reports of assessing client eligibility for unsupervised self-injection of DMPA-SC varied. From the provider's perspective, requirements for clients to prove their competence with self-injection were typically rooted in a desire to maintain fidelity and support client safety. However, such variation in the refill-seeking process can present a barrier to clients accessing their preferred contraceptive method. Some clients may have to repeat steps in the path to unsupervised self-injection at the point of seeking refills of DMPA-SC, such as receiving a provider-administered dose, and/or doing supervised self-injection, before being allowed to resume unsupervised self-injection. Other clients may choose one SDP over another based on less stringent requirements to obtain a refill of DMPA-SC, or even opt to pay for units from a private SDP to avoid setbacks.

Reliable access to DMPA-SC for unsupervised self-injection was further impacted by stockouts of the method, an ongoing concern since the implementation of the method in Nigeria (27). IDI respondents spoke about rationing units of DMPA-SC dispensed to clients out of concern for future stockout and to ensure they had enough units for other clients who may ask for the method. Stockouts also led some respondents to purchase DMPA-SC themselves on the open market and transfer the cost of the method onto the client. This temporary fix negatively impacted both providers and clients: providers spent their own time and money to maintain a supply of DMPA-SC, and clients faced the undue burden of occasionally paying for a product they might expect to be free from the government. While it is expected that private SDPs sell contraceptives, the provision of free contraception from public SDPs was inconsistent and varied by state. Namely, public sector providers in Enugu and Plateau often had to purchase these items out of pocket and charge clients for the products to recover costs.

As DMPA-SC for self-injection has been implemented via a total market approach in Nigeria, it was important for us to include both public and private sectors in our sample and consider how the experience of offering and accessing the method differed between the two sectors. One conclusion that we can draw from our descriptive findings is that stark differences in the DMPA-SC care-seeking experience between the two sectors were minimal. In both public and private SDPs, MC actors and healthcare providers reported a variety of criteria used to assess clients' eligibility for unsupervised self-injection, issues with DMPA-SC stockouts, and different approaches to dispensing refills of DMPA-SC to self-injecting clients. To understand implementation holistically, it is important to document when there are no differences.

The results from this descriptive analysis should be considered alongside several limitations. First, MC actors' reports of judgment from providers and retailers were subjective and should not be interpreted as definitive indications of provider bias. We extensively trained actors to standardize visits and post-visit reports, but reports are still subject to individual perception and interpretation. Second, while MC methodology offers the advantage of controlling some variables of an interaction, it limits the generalizability of findings as only a specific scenario/context is presented by the MC actor. Thus, certain complex scenarios that real-life providers and clients face were not captured. Family planning clients have a broad spectrum of experiences, intersectional identities, and preferences that cannot be captured in MC interactions. Third, the actors specifically sought DMPA-SC for self-injection, both as new users and continuing users of the method, which may have affected providers' recommendations for and against other methods if the provider-client conversation was focused on DMPA-SC. While we have data on the methods that providers recommended to the actors, reasons for recommendations were not captured in this data, and we cannot speak to the rationale behind method recommendations. Finally, while the selection of three states in Nigeria (Enugu, Lagos, and Plateau) offered some degree of urban and rural representation, as well as varied stages in the scale-up of DMPA-SC for self-injection, these areas cannot fully represent the provider perspectives and behaviors across the country. The DMPA-SC for self-injection care-seeking experience is likely to be different in other states, and even LGAs within the study states, based on factors such as rurality, conflict, predominant religion in the area, and more.

## Conclusion

This descriptive study aimed to explore the experiences of initiating and continuing DMPA-SC for self-injection in Nigeria, including identification of gaps in meeting the needs of both clients and providers, by using a combination of MC interactions and qualitative interviews with family planning providers to capture a range of perspectives on access and provision across different regions of the country. These observations provide a unique contribution to the literature on implementation of DMPA-SC for self-injection in Nigeria, by incorporating both provider and simulated client perspectives on method continuation and the refill-seeking process, which has been a noted gap in previous research.

Our finding that clients seeking refills of DMPA-SC to continue unsupervised use may face setbacks in their self-injection initiation process highlights the challenges that the multi-step self-injection initiation protocol places on both clients and providers. Providers spoke about requiring hypothetical clients, who claimed to have experience with unsupervised self-injection of DMPA-SC, to demonstrate their competence via provider-supervised self-injection, or receiving a provider-administered dose before resuming unsupervised self-injection. Such requirements, while seemingly rooted in desire to maintain fidelity and support client safety, lead providers and clients to miss out on potential benefits of self-care via self-injected contraception, such as reducing provider workload and increasing client autonomy (1). To address this, guidelines should offer providers a set of acceptable options to assess eligibility for unsupervised self-injection, as well as a clear recommendation on the number of units to dispense per refill. In addition, a consistent and reliable supply of DMPA-SC in SDPs across Nigeria is crucial to ensuring predictable dispensing patterns.

Providers carry a great deal of responsibility in ensuring equitable access to self-care contraception, such as self-injectable DMPA-SC. Clear guidelines for providers will aid in accomplishing this, but wider systems interventions are also necessary. Interventions that address norms about what types of clients are well-suited to self-inject, simplify refill protocols, and ensure consistent method supply are also critical to creating an enabling environment for providers and clients to access self-care contraceptives.

## Data Availability

In line with the grant agreement, quantitative data will be made publicly accessible six months after publication of the results in a peer-reviewed journal. Qualitative transcripts will not be made publicly available due to the need to ensure a high degree of respondent confidentiality.

## Footnotes

^1^Although continuation of a method is not inherently a good outcome - since it may result from coercion by a partner, provider, or other external influence - it can serve as a proxy for a client's ability to manage their contraceptive use in accordance with their preferences.

^2^Four critical steps to SI: (1) Mix the solution by shaking vigorously for 30 seconds. (2) Push the needle cap and port together to activate the device. (3) Pinch the skin to form a “tent” and insert the needle. (4) Squeeze the reservoir slowly, for 5 to 7 seconds, to inject the contraceptive.
